# Processing Next-Generation Mass Spectrometry Imaging Data: Principal Component Analysis at Scale

**DOI:** 10.1021/jasms.4c00314

**Published:** 2024-10-28

**Authors:** Kasper Krijnen, Paul Blenkinsopp, Ron M. A. Heeren, Ian G. M. Anthony

**Affiliations:** † The Maastricht MultiModal Molecular Imaging Institute (M4i), Division of Imaging Mass Spectrometry, Maastricht University, Maastricht 6229 ER, The Netherlands; ‡ Ionoptika Ltd., Unit B6, Millbrook Cl, Chandler’s Ford, Eastleigh, SO53 4BZ, United Kingdom

**Keywords:** mass spectrometry imaging, random access memory, principal component analysis, incremental principal component analysis, Python, algorithm

## Abstract

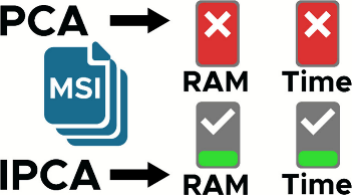

Mass spectrometry imaging (MSI) is constantly improving in spatial resolving power, throughput and mass resolution. Although beneficial, these improvements increase data set size and content. The larger data requires correspondingly fast computer-based analyses. However, these analyses often do not scale well with increased data size. Principal component analysis (PCA) is an important analytical tool commonly used with MSI data; however, most PCA algorithms load and process the entire data set within random access memory (RAM) which is most often insufficient for large data sets. PCA algorithms that use less RAM than the data set exist but are usually much slower or sacrifice precision and are rarely used for MSI data processing. Incremental PCA (IPCA) is an alternative algorithm that avoids large RAM allocations while also preserving speed and analytical precision. Here, we demonstrate and benchmark the use of differing implementations of IPCA, PCA, and commercial software on large and often complex MSI data sets. We show that using an already-published Python-based IPCA algorithm, IPCA can be successfully applied to MSI data sets too large to fit with RAM. Furthermore, our benchmarks demonstrate that, contrary to expectations, IPCA is faster than all other tested PCA implementations on all large data sets that can be directly compared.

## Introduction

Many improvements in mass spectrometry imaging (MSI) instrumentation are focused on the improvement of throughput,^1,2^ spatial resolution,^3−5^ sensitivity,^4,5^ and mass resolution.^6^ Although beneficial, these improvements produce increasingly complex and large data. This data requires automated and semiautomated tools to highlight relevant spatial areas and mass peaks as well as to reduce complexity. One of the most used MSI data analysis techniques is principal component analysis (PCA). PCA is used to perform dimensionality reduction and is often a precursor to more sophisticated data analysis routines, such as classification,^7^ segmentation,^8^ or other dimensionality reduction techniques such as NMF, t-SNE and UMAP.^9−11^ Because of the ability to reduce the data size, PCA is often a starting point for exploratory analyses and data analysis approaches of large data sets, even if more sophisticated dimensionality reduction is utilized for the final workflow. As such, the inability to perform PCA on very large data sets eliminates the use of many other algorithms later in the processing pipeline.

Popular programs for routine MSI data analysis that can perform PCA are SCiLS Lab^12^ and LipostarMSI.^13^ Other options exist such as MSiReader^14,15^ or the R package Cardinal.^16^ Although these implementations provide many tools for the analysis of data, for PCA most of these tools load the entire data set into random-access memory (RAM). For data sets somewhat larger than the computer’s store of RAM, portions of the data that would have otherwise been loaded into RAM can be stored on the drive in virtual memory temporarily. However, such temporary storage can slow data processing. When performing PCA with data sets that are, for example, more than double the size of RAM, a user has only two options: increase the available RAM or reduce the size of the data set by e.g. binning or truncation. Besides the respective drawbacks of increased costs and reduced accuracy, these solutions cannot scale indefinitely.

The combination of technological improvements in mass resolution, spatial resolution, sensitivity, and throughput have caused MSI data size and complexity to outpace computer hardware improvements. Thus, solutions for the analysis of very large, detailed MSI images or data sets of multiple MSI images are needed that use improved data processing algorithms. One such improvement is to adapt PCA to a series of steps that can each be completed with a fixed amount of RAM, such that the slowdown caused by disk-based virtual memory is never encountered. Such RAM-efficient PCA methods usually come at the cost of reduced speed when applied to data sets that fit within RAM or reduced accuracy. Examples of such methods include online PCA,^17^ Randomization,^18,19^ and Incremental PCA (IPCA).^20−22^ These high-RAM efficiency methods have been employed in a limited fashion for the analysis of MSI data in the past.^23,24^ However, such algorithms are not commonly used for MSI data. Unlike other such algorithms, IPCA retains high accuracy^20,21^ making it an attractive option for general replacement of PCA for MSI data sets if the performance costs can be successfully mitigated.

Herein, we present and benchmark incremental PCA (IPCA) implementations based on the Python package sklearn’s^22^ IPCA algorithm that was optimized for use with large MSI data sets. We compare the optimized IPCA against both standard PCA functions as well as commercial software. We demonstrate that, as expected, IPCA can be successfully applied to large MSI data that cannot be processed with conventional PCA implementations. Furthermore, we show that, contrary to expectations, IPCA can perform orders of magnitude faster than commercial software and even faster than comparable PCA on data sets that fit entirely within RAM.

## Methods and Materials

### Samples for Data Sets

Five data sets (I–V) were used for benchmarking. (I) The mouse bladder data set^25^ is a publicly available data set in the PRIDE Database.^26^ (II) A cancer xenograft was obtained from Johns Hopkins University under ethical approval (2014–108 at GROW Maastricht University and A3272–01 at the Johns Hopkins University) for a different study.^27^ An unused and unimaged serial section of this xenograft was used for this study. (III) Spinal columns of rats were acquired for a different study (project license 2017–022 Maastricht University) and imaged with MSI. (IV) MSI images of multiple livers were acquired for a different study that has not yet been published. (V) A large-area MSI image of a composed surface was acquired for a different study that has not yet been published. This composed surface contained a slice of lemon (from a local grocer, sectioned using a cryostat, Leica Biosystems, Wetzlar, DE), two TEM grids (300 mesh thin-bar copper and 300 mesh hexagonal thin bar nickel, Agar Scientific LTD), spotted solutions of gadolinium and holmium chloride hexahydrate (Sigma-Aldrich, St. Louis, USA), red phosphorus, and a fingerprint of a solution of table salt in water.

### Instruments

The “Cancer Xenograft” (II) image was acquired with a J105 mass spectrometer (Ionoptika, Ltd., Chander’s Ford, UK) equipped with a gas cluster ion beam (GCIB) model GCIB SM. Water cluster sizes of 30 000 were used at an energy of 60 kV and with a spatial resolution of 16 μm. The “Spinal Columns” (III) images were acquired using a rapifleX (Bruker Daltonics GmbH & Co. KG, Bremen, DE) with a DHB matrix at 5 μm spatial resolution. The “Multiple Livers” (IV) images were acquired using a timsTOF fleX using MALDI-2 (Bruker Daltonics GmbH) with Norharmane matrix at 10 μm spatial resolution. The “Composed Surface” (V) image was acquired on a customized “BioTRIFT” Instrument equipped with a TPX3CAM (Amsterdam Scientific Instruments, Inc., Amsterdam, NL) and a C60^+^ ion gun (Ionoptika, Ltd., Chander’s Ford, UK). Seven acquisition passes of the composed sample were acquired using a previously described method with a pixel size of 2 μm.^2^ These seven images were summed to produce a single mass image with more ion counts than an individual image. The 33 most abundant peaks were selected and processed into a datacube specifically for this study.

### Computer Hardware

All benchmarking was performed on a desktop personal computer containing an Intel i7–10700K CPU (3.8 GHz with boost to 5.10 GHz, 8-core, 16 thread), 64GB RAM (DDR4 at 2933 MHz) and a 1TB SSD (model MZVLB1T0HBLR-00H1, Samsung, KR).

### Software

MSI data analysis software SCiLS Lab (version 2023c/Release 11.02, Bruker Daltonics GmbH)^7^ and LipostarMSI (version 2.0.18, Molecular Horizon srl, Bettona, IT) were used for benchmarking. Additionally, the R package Cardinal^16^ is used for MSI data analysis and includes a highly RAM efficient PCA function from the IRLBA package that was used for comparison.

These commercial software and R package were compared against custom scripts consisting of (A) a Python-based IPCA algorithm (modified from an implementation in scikit-learn), (B) A Python-based PCA algorithm that randomly sampled 6.5% of the pixels and calculated loading scores from this subsample (C) a Rust-based PCA algorithm, (D) a Rust-based IPCA algorithm optimized for RAM utilization, (E) a Rust-based IPCA algorithm optimized for multithreaded performance. The Rust-based implementations of IPCA were based on the modified scikit-learn implementation of the IPCA algorithm.

Rust (2021 edition, version 1.78.0-nightly) and Python (CPython version 3.9.13) were used with the multithreaded singular value decomposition (SVD) function from the Intel oneAPI Math Kernel Library (version 2023.1, settings: static, lp64, iomp).

### Data Preprocessing

Depending on the data set, a slightly different preprocessing approach was used due to differences in starting data file format and layout. The general approach consisted of (A) loading the data into SCiLS Lab, (B) peak picking using SCiLS Lab or the SCiLS Lab API, and (C) exporting the peak list and peak picked data. When benchmarking SCiLS Lab, step (C) was omitted in favor of directly performing PCA. For LipostarMSI and R Cardinal, the data were separately converted to the imzML file format; then both the exported peak list and converted data were imported and used for PCA. For Python and Rust (I)PCA, the exported data was converted to a binary datacube and the exported peak list and metadata were converted to a corresponding JSON file. The binary datacube consisted of a “flat” series of 32-bit IEEE-754 floating point numbers for improved vectorization, with a size of *m* × *n* × 4 bytes, where *m* is total number of spectra and *n* the number of peaks. Metadata included spatial labels (e.g., regions for data sets including multiple images), information regarding offsets per region, and total numbers of pixels and peaks.

The general approach was adjusted for the “Spinal Columns” data set. This data set contained multiple large images where only a smaller region of interest was intended for analysis; thus, the data set was much larger than all other data sets before preprocessing and cropping away the unwanted regions. The complete data set was not able to be loaded into either LipostarMSI and SCiLS Lab. Peak picking was performed separately for each of the small regions and combined manually into one peak list with the SCiLS Lab API, after which step C of the general approach could be used.

Not all data sets were benchmarked by all PCA implementations. Specifically, SCiLS Lab was unable to load the complete “Spinal Columns” data set; however, individual MSI images could be loaded to permit the workflow above. Similarly, LipostarMSI was unable to load either the “Spinal Columns” or “Multiple Livers” data sets. Finally, neither SCiLS Lab nor Lipostar were able to load the “Composed Surface” data set due to its large size and number of pixels (>250 million; which would be ∼250 GB if converted to .imzML).

### Benchmarking

A Python script was executed before the start of each benchmarking test to record the RAM utilized for each PCA or IPCA process at sampling intervals of 15 ms. Processing time was also measured using this Python script for LipostarMSI and SCiLS Lab. This processing time was defined by the increase in RAM usage from the resting RAM, until dropping to a new stable resting RAM usage after PCA. This method of benchmarking allowed for more precise and consistent timing than the times reported by either LipostarMSI or SCiLS Lab. LipostarMSI and SCiLS Lab were restarted for each benchmark measurement. Additionally, a “resting” average RAM was acquired for these programs before starting the PCA processing. For the reported values of the RAM utilization, the resting average RAM was subtracted from the peak RAM.

Internal timing variables were used for measuring processing time in the R, Rust, and Python (I)PCA implementations. The internal timing variables were placed in the code to measure the time of batch size calculation, creation of the memory map, (I)PCA, and saving the principal component values to a binary file.

All benchmarks for the five data sets were performed in ten replicates, except for the LipostarMSI benchmark for the Cancer Xenograft data set which was performed in triplicate due to long run times.

The internal PCA functions of the LipostarMSI and SCiLS Lab software packages may change with later versions. Their inclusion in this study is to provide a “snapshot” of MSI workflows currently in use.

### IPCA Workflow

The Python and Rust (I)PCA implementations with instructions for use with an example data set has been made available on GitHub at: https://github.com/KKrijnen/IPCA. The IPCA algorithm is a modification of the scikit-learn Python library function IncrementalPCA which is based on Sequential Karhunen-Loeve.^20,21^ For the fitting part of the algorithm, the time complexity for each iteration is *O(dm*^*2*^*)* with space complexity of *O(dm)*(20,22) Where *d* is the batch size and *m* is the number of features (mass peaks). Figure S1 provides measurements on the memory and runtime of IPCA on randomly generated data matrices.

**Figure 1:**
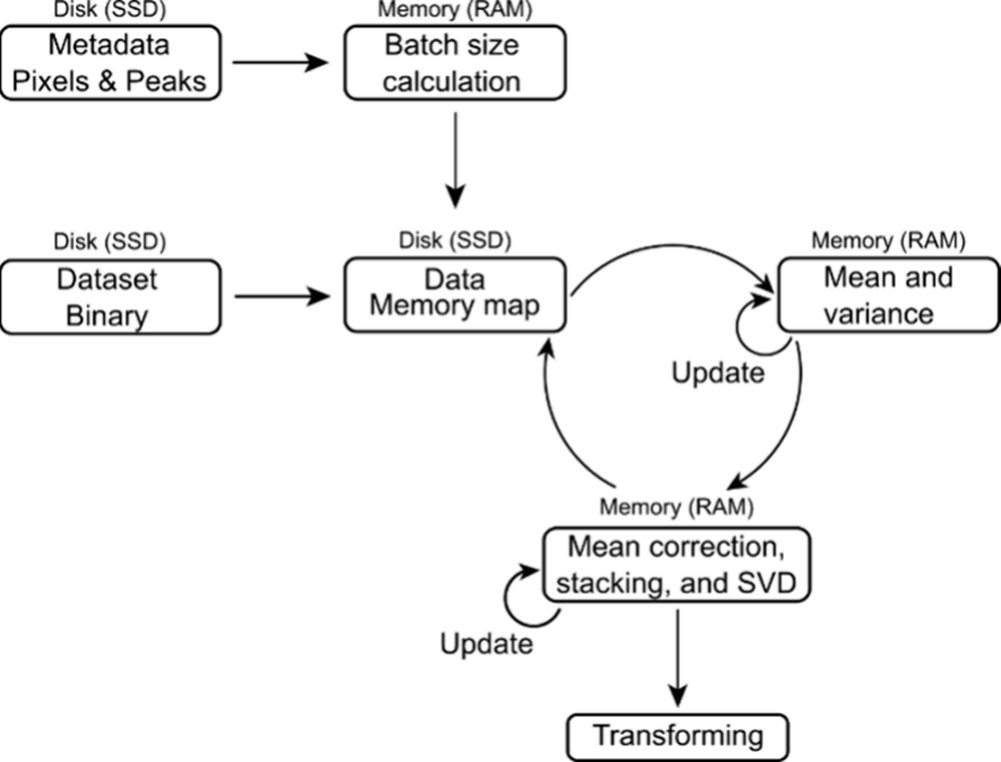
IPCA workflow begins with constructing a memory map, loading metadata, and calculating optimal batch sizes. A portion of the data are loaded into RAM. The mean and variance are calculated and a running mean is updated. SVD is applied and a running set of singular values are updated. The cycle then repeats until all data has been processed. At that point a transformation (dot product) is applied to produce the principal components.

The scikit-learn IncrementalPCA function was optimized for MSI data by (I) modifying the function to ensure use of 32-bit floating point numbers instead of 64-bit floating point numbers. This modification enables both less RAM utilization as well as faster vectorized processing; (II) adding a batch size calculation function that determines how many mass spectra (i.e., pixels) each IPCA loop should process — this was heuristically determined to be roughly 19× the number of mass peaks (i.e., features). (III) Performing the final transformation step (shown in Figure 1) in an RAM-efficient incremental fashion rather than in a single step.

Figure 1 illustrates the IPCA algorithm employed in both the Rust and Python implementations. Pseudocode and a brief description of the IPCA algorithm may be found in Figure S2. The algorithm starts with a memory map being generated for the data set. Memory maps allow loading a small portion of the data into RAM at a time, rather than loading the entire data set at once. Next, the batch size to be loaded is estimated. Utilizing the memory map, a single batch is loaded from the SSD into RAM (Figure S2, line 8). The mean and variance of the data set are updated incrementally to represent the mean and variance of all batches up to the current batch (Figure S2, lines 11 and 13). The mean of the current batch is subtracted from each peak (Figure S2, line 12), this mean corrected data is stacked vertically with the results of the previous iteration to produce a matrix (Figure S2, line 18). This matrix serves as input for the next SVD (Figure S2, line 20). The SVD results are updated, and the process repeats until all data is processed (Figure S2, line 26). Importantly, the memory map should be closed and reopened at each iteration. Closing and reopening a memory map is uncommon; however, if not closed then the previously loaded data is not dropped from memory. In practice, such closing and reopening was observed to be faster than using a buffered reader approach. In the final batch, the explained variance is calculated, and the loading scores are saved (not shown in pseudocode). More detailed descriptions of this algorithmic process, including optimizations and alternate methods of performing steps, are described elsewhere.^15,16^

After fitting, the data is transformed by multiplying the mean-subtracted original data with the loading scores from the fitting phase to obtain the principal component scores. This transformation step (shown in Figure S3) is performed in increments. A segment of the data is loaded into RAM using the memory map, and the dot product with the loading scores is calculated and stored on the SSD. This process is repeated for each subsequent batch until the component values for the entire data set are obtained.

## Results and Discussion

Table 1 shows benchmarking results of analysis using six methods (under the “Algorithm” column) of performing PCA on five data sets. The size of each data set is provided as the number of spectra × number of mass peaks. The rounded size of the datacube is provided below these numbers, for example, for the Cancer Xenograft data set, the data size is 262 144 × 310, which when provided as 32-bit floating point numbers results in a size of 325 MB. All trials except those using R IRLBA C3 and C30 had a standard deviation of less than 5%. Exact deviation values, additional precision, full data set size, and ratios of RAM used to datacube size are provided in Table S1. RAM usage was measured as the peak of RAM usage during the (I)PCA, minus the RAM before the start of (I)PCA. This is to ensure that only RAM for the PCA itself is measured and not other background processes or, e.g., graphical user interface elements.

**Table 1:** Benchmarking Results of (I)PCA

	Mouse Blader	Cancer Xenograft	Spinal Columns	Multiple Livers	Composed Surface
Mass spectra (× 10^6^)	0.035	0.26	0.57	0.82	256.67
Mass Peaks	199	310	415	573	33
Datacube Size (MB)	28	325	949	1873	33886
Algorithm	Time (s)				
LipostarMSI	252	6 740	a	a	a
SCiLS Lab	1.76	37.5	a	237	a
R IRLBA C3	16.0	181	488	964	a
R IRLBA C30	77.4	792	1960	3630	a
6.5% Sampled	0.0878	0.873	2.76	5.60	151
Python IPCA	0.168	2.38	7.48	17.0	252
Rust PCA	0.138	3.47	10.9	23.5	b
Rust IPCA	0.171	1.89	5.74	13.2	188
Rust IPCA^d^	0.152	1.58	4.90	11.2	141
Algorithm	RAM (MB)				
LipostarMSI	102	990	a	a	a
SCiLS Lab	567	5 870	a	32 900	a
R IRLBA C3	97.4^c^	313^c^	562^c^	978^c^	a
R IRLBA C30	89.8^c^	548^c^	1045^c^	1310^c^	a
6.5% Sampled	28	331	970	1911	35697
Python IPCA	52.8	155	157	158	216
Rust PCA	80.4	1060	3 010	5 780	b
Rust IPCA	53.0	154	154	154	153
Rust IPCA^d^	53.2^d^	615^d^	1730^d^	2450^d^	2 490^d^

a Unable to be loaded.

b Out of memory error.

c Large standard deviation due to R garbage collector.

d With a multithreaded transformation loop.

### Commercial Software

In both LipostarMSI and SCiLS Lab there is a limit to how many components can be selected for PCA. For LipostarMSI and SCiLS Lab this limit is 99 and 100 components, respectively. The Python and Rust (I)PCA implementations calculate all components. This is equal to the number of features (mass peaks) in each input data set. As can be observed in the table, LipostarMSI utilizes a RAM-efficient, although time-inefficient algorithm for performing PCA. It is hundreds to thousands of times slower than the other PCA implementations. In practice, lowering the number of principal components to be calculated decreases run-time. SCiLS Lab was approximately 10 to 20 times slower than the Rust and Python (I)PCA implementations. SCiLS Lab also required the most RAM of any of the PCA workflows, requiring 33 000 MB RAM for the Multiple Livers data set. As mentioned in the methods section, SCiLS Lab, LipostarMSI, and Cardinal were unable to load some data sets (denoted with a †) due to lack of available RAM. Due to the R garbage collection occurring during the IRLBA-based PCA, the variance of utilized RAM was higher than with other PCA methods (noted with a §)

### R Cardinal IRLBA PCA

The R package Cardinal utilized the IRLBA package for PCA to allow for memory-efficient processing of MSI data. As can be seen in Table 1, although memory efficient, the speed of the IRLBA-based PCA was substantially lower than that of most other algorithms. Cardinal allows for selecting a lower number of PCs to be computed than the feature size. Decreasing this number can increase speed. As such, the IRLBA-based PCA was performed with both 3 and 30 PCs selected. Selecting the same number of components to identify as the number of features (e.g., 573 for the “Multiple Livers” data set) causes errors in many cases and is not recommended.

### 6.5% Sampled PCA

Sampling a small (6.5%) portion of the pixels and calculating loading values from this subsample allowed for a corresponding speedup and is faster than all other approaches generally. Additionally, the subsampling approach yielded competitive RAM utilization. However, such RAM utilization scales linearly with the size of the data. Such a linear scaling is observable in Table S1 where the RAM utilized is divided by the data cube size, and the subsampled PCA approach is constant. Despite being superior in runtime and requiring less RAM than naïve PCA, the subsampled approach cannot scale infinitely with data size. This may be observed in Table 1 where the 6.5% sampled approach was tested using the “Composed surface”. Although able to be performed, this required substantial amounts of RAM and with a larger data set (e.g., one with 90 features instead of 33) would not have been possible to analyze.

### Rust-Based (I)PCA

The naïve Rust PCA approach was faster than LipostarMSI and SCiLS Lab and required less RAM than SCiLS Lab while requiring comparable RAM to LipostarMSI. This was to be expected as the structure of the data file was known *a priori* in the Rust PCA implementation and thus optimizations such as linear memory maps and the use of 32-bit floating point numbers could be used throughout the implementation. Despite these advantages, the composed surface data set was impossible to analyze with naïve PCA as naïve PCA required more RAM than installed on the computer system. Surprisingly, the naïve Rust PCA was slightly slower than all IPCA implementations even though IPCA in principle requires more computations to complete. We attribute this surprising result to the optimizable parameter that allowed for selection of batch size in IPCA but not in PCA. This batch size selection in IPCA may enable optimizations during the SVD step. Figures S4 and S5 provide details on effects of batch size on runtime and discussion of batch size choice that was heuristically determined. Additionally, loading a smaller batch rather than the entire data set may have allowed for more cache-friendly loading and fewer RAM-based bottlenecks.

The Rust IPCA implementation (with single threaded transformation loop) was ∼1–1.3 times faster than the Python IPCA implementation and was up to ∼1.3 times faster still by multithreading the transformation loop. Albeit this transformation step came at the cost of requiring extra RAM proportional to the number of threads utilized for the data set. Thus, the RAM utilized in Table 1 for the multithreaded Rust IPCA is a reflection not on the full IPCA algorithm but on the final transformation step.

### Python Based IPCA

The Rust-based IPCA implementation was written and tested to measure the improvement in processing time due to the generally higher performance of Rust-based software compared to Python. Although, the Rust-based IPCA performed substantially better in both speed and RAM use than the naïve Python-based IPCA (Table S1) it was noted that the optimizations for the Rust-based IPCA workflow could be applied to the Python scikit-learn IPCA workflow. With these modifications, the Python IPCA function was only between ∼1–1.3 times slower than the single threaded transformation loop Rust implementation and ∼1.1–1.8 times slower than the multithreaded transformation loop Rust implementation. The RAM usage of the Python IPCA implementation was similar to the single threaded Rust IPCA implementation.

### Performance and Recommendations

The performance similarity between the Rust and Python IPCA implementations is surprisingly close, given the disparity in speed between the two languages where Rust is usually orders of magnitude faster. This similarity is explainable because most of the computations are performed by the same MKL functions in both the Rust and Python IPCA implementations. Thus, it is expected that similar performance would be achieved by any other language (e.g., MATLAB, Julia, R, etc.) that makes use of these MKL functions. Based on these results, the recommendation for readers who wish to apply IPCA to their MSI data processing workflows is to use Python and apply the three relatively straightforward optimizations used for the scikit-learn IPCA function. These optimizations are discussed in the IPCA Workflow section (as I–III) and detailed in the https://github.com/KKrijnen/IPCA repository. This recommendation is because scikit-learn provides documentation and ease-of-use features such as automatic linking with the relevant MLK and can be used in combination with the SCiLS Lab API and other easy-to-use Python libraries.

### Visual Inspection and Accuracy of IPCA

**Figure 2:**
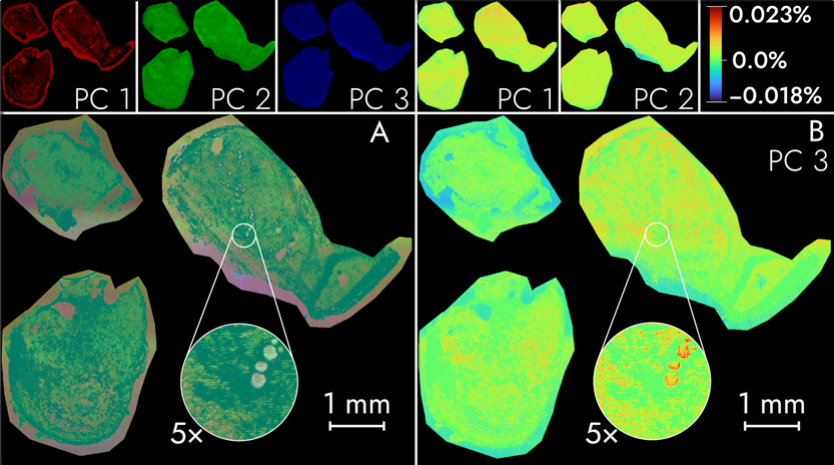
Hyperspectral visualization of the first three principal components for the Multiple Livers data set processed by Rust IPCA (A) and compared against the processed results using SCiLS Lab using difference heatmaps (right panel). Principal components 1, 2, and 3 are visualized as linear gradients of red, green, and blue, respectively in the left panel. The maximum difference between the SCiLS lab and IPCA based results are observed for PC3 (B) and are between −0.018% and +0.023% of the range between the minimum and maximum PC3 Rust IPCA-produced pixel.

Figure 2 shows a hyperspectral image of the first three principal components of the Multiple Livers data set as produced by the Rust IPCA implementation (A). The outputs between the Rust IPCA and the SCiLS lab PCA results are very nearly identical. This may be observed in the very low differences that are shown in the right panel. Figure S6 provides a side-by-side comparison of the specific Rust IPCA and SCiLS lab PCA results. These results highlight the benefits in accuracy of the IPCA approach over other high speed and RAM-efficient PCA approaches that may sacrifice accuracy, such as calculation of PCA loading scores by randomly sampling a subset of pixels.^24^

Table S2 contains mean squared error (MSE) values of the loading scores of SCiLS lab against Rust IPCA, R IRLBA C30, and 6.5% sampled PCA. The 6.5% sampled PCA results show consistently higher MSEs than the Rust IPCA and R IRLBA C30, which are nearly identical. The coefficient of determination (r^2^) between the respective SCiLS Lab and Rust IPCA component loading scores for the Multiple Livers data set provide a metric of determining the similarity between these two methods (Table S3). The initial ten loading scores from SCiLS Lab have an average r^2^ of 0.999 with the corresponding Rust IPCA loading scores. Together, these ten loading scores account for 99.858% of the total variance (for both Rust IPCA and SCiLS Lab). For components 11 to 20, which account for 0.070% of the variance, the average r^2^ between the SCiLS Lab and Rust IPCA is only 0.99. The average r^2^ drops to 0.98 for components 21 to 30. Similar to the MSE comparisons, the Rust IPCA and R IRLBA C30 results were highly similar to both SCiLS Lab and to one another. The 6.5% sampled PCA results were substantially different from other algorithms — especially after PC11. As the explained variance by the components decreases, the corresponding amount of agreement between the (I)PCA implementations decreases; some of this decrease in r^2^ can be attributed to 32-bit floating point error as r^2^ improved when a preliminary test was performed using 64-bit floating point numbers. In most MSI data analysis routines, principal components that do not explain much variance, for example those after the first 30, are not generally used; this is one reason that SCiLS Lab and LipstarMSI do not allow selection of more than 100 components.

A somewhat surprising finding was that, although poorer in quality, the subsampled PCA results of 6.5% of the total pixels were less poor than expected within the first approximately 11 PCs for the specific results of the Multiple Livers data set. Although the subsampling approach has a drawback of not providing a complete solution to the scaling problem of MSI data, it is important to note that it is not exclusive with nonincremental PCA. An even faster solution for some applications may thus be combining randomized subsampled PCA with IPCA. An interesting advantage of this hypothetical approach is that it could be performed on increasing percentages; thus the algorithm could be “stopped” when a convergent threshold or time limit had been reached.

### Increases in MSI Data Set Size

**Figure 3:**
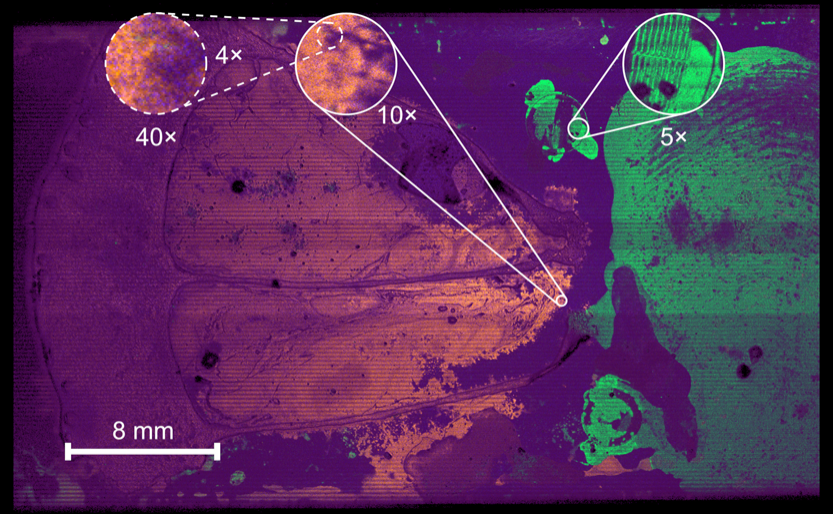
Hyperspectral image of the first three components (mapped to red, green, and blue color channels for components 1–3, respectively) of the Composed Surface data set. The original data set contains 256 711021 pixels and 33 mass peaks (i.e., features) with a pixel size of 2 μm.

Figure 3 shows the first three components of the Composed Surface data set. The Composed Surface data set is a large-enough image (33 886 MB when processed into a datacube binary file containing 32-bit float numbers) that the 64 GB of RAM of the computer used for benchmarking was insufficient for naïve PCA, which when attempted caused an “out of memory” error. This data set is an example of new, high-throughput MSI data that demonstrates the importance of improving PCA runtime and RAM utilization as the MSI imaging data for this data set were collected in less than 45 min. The ∼34 GB datacube was the result of centroiding the original data to only 33 peaks (out of ∼30,000 mass channels). If processed in profile-mode, the uncompressed datacube would be more than 28 TB. The use of only 33 peaks represents aggressive data culling and is one strategy to improve PCA performance. However, even such strategies are not sufficient to keep pace with the rapid rate of MSI data size expansion. For instance, the composed surface was a single image, however serial images of tissue sections can mean that a single data set produced in a single a workday of imaging could be several hundred GB or even TB of data; even when stored in an efficient centroided format. Due to the large size of the image, only the IPCA implementations were able to analyze the image.

## Conclusion

Advancements in mass spectrometry imaging, particularly in throughput, spatial resolution, sensitivity, and mass resolution, have led to the generation of increasingly large and complex data sets, necessitating efficient data processing algorithms. Here we investigated optimized implementations of an IPCA algorithm for mass spectrometry imaging data and provides information for utilizing IPCA libraries with large MSI data sets. Furthermore, the IPCA workflow was validated on MSI data sets against PCA.IPCA could analyze a large data set (>250 million pixels; 33 features) that PCA was incapable of analyzing due to out of memory errors. Surprisingly, IPCA performed faster than PCA for most data sets that could be analyzed with both IPCA and PCA equally. We anticipate the use of IPCA for both routine studies as well as for future large MSI studies that use instruments with high throughput and mass resolution.

## Supporting Information Available

Graphs of performance of IPCA on randomly generated data sets, pseudocode of the IPCA function, extended workflow of the transformation step of IPCA, table of detailed benchmarking results, graphs of the effects of batch size on runtime, graphs of the effects of batch size on runtime of a real data set, table of the mean squared errors of algorithms compared to SCiLS Lab results, table of the r^2^ of algorithms compared to SCiLS lab results (PDF)
