# Supplementary material for: Processing Next-Generation Mass Spectrometry Imaging Data: Principal Component Analysis at Scale
Source: J Am Soc Mass Spectrom. 2024 Oct 28;35(12):3063–9. doi: 10.1021/jasms.4c00314 (PMC11622226; doi:10.1021/jasms.4c00314)
Supplement: Supplementary file 1 — js4c00314_si_001.pdf [file js4c00314_si_001.pdf]

Supporting Information:

Processing next-generation mass spectrometry imaging data: principal component analysis at scale

Kasper Krijnen[a], Paul Blenkinsopp[b], Ron M. A. Heeren[a], Ian G. M. Anthony[a]*

[a]The Maastricht MultiModal Molecular Imaging Institute (M4i), Division of Imaging Mass Spectrometry, Maastricht University, Maastricht 6229 ER, The Netherlands, [b]Ionoptika Ltd., Unit B6, Millbrook Cl, Chandler's Ford, Eastleigh, SO53 4BZ UK

Contents

Figure S1: Performance of IPCA on randomly generated matrices at different sizes 2

Figure S2: Incremental PCA Pseudocode 3

Figure S3: Transformation step workflow... 4

Table S1: Additional detail for benchmarking results 5

Figure S4: Batch size optimization threshold.. 7

Figure S5: Batch size optimization threshold applied to a "real" dataset 8

Figure S6: Visual comparison of SCiLS and Rust IPCA results 9

Table S2: MSE values of the comparison of algorithms compared against SCiLS Lab loading scores for the Multiple Livers dataset 10

Table S3: r^2 values of the comparison of algorithms compared against SCiLS Lab loading scores for the Multiple Livers data 11

IPCA Repository: https://github.com/KKrijnen/IPCA

*i.anthony@maastrichtuniversity.nl

Figure S1: Performance of IPCA on randomly generated matrices at different sizes

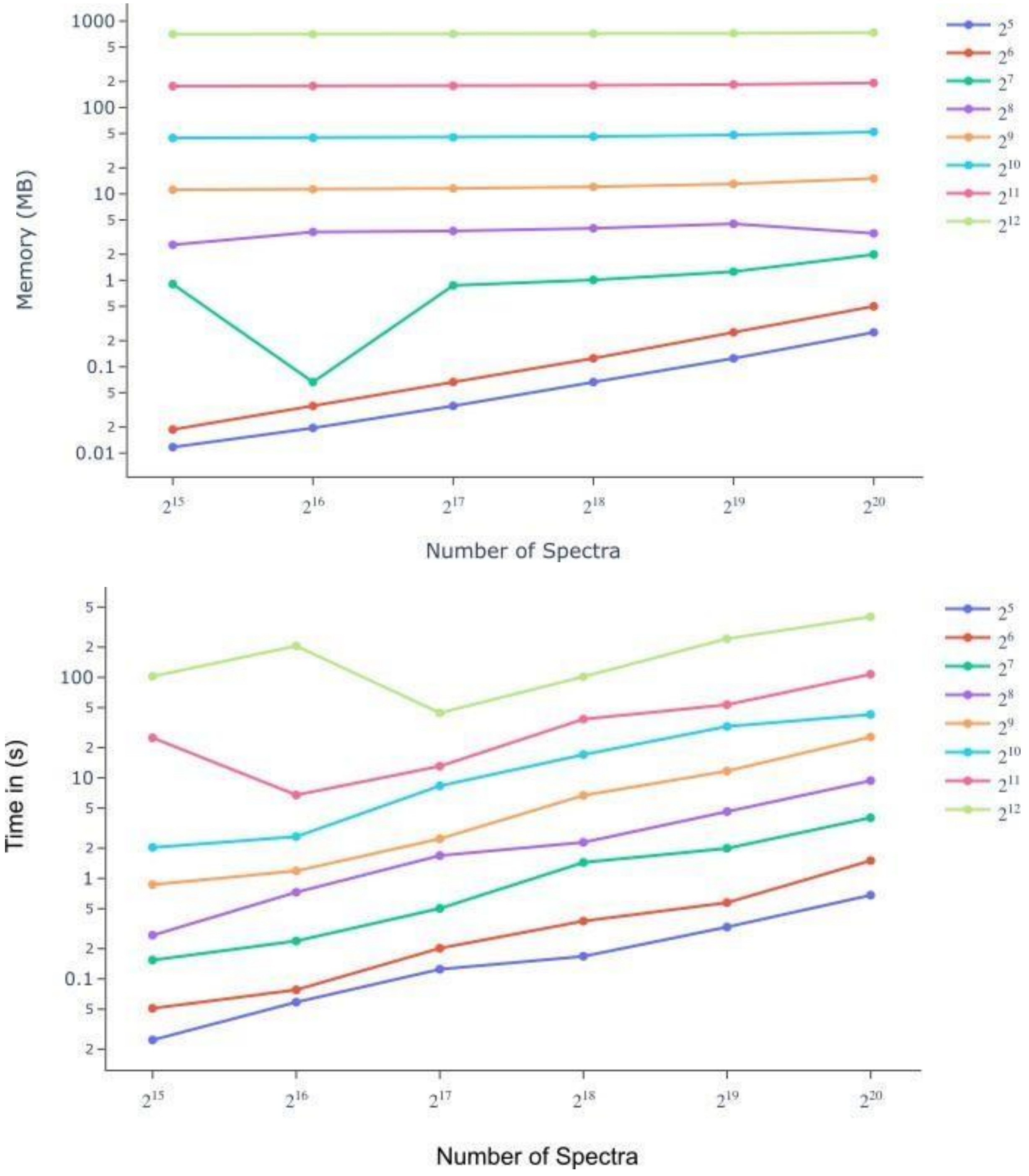

Figure S1: Performance of the Rust-based IPCA algorithm on matrices of randomly generated numbers which demonstrate the increase in RAM utilization (top) and runtime (bottom) as a function of dataset size. Of note is that the runtime scales proportionally (linear on the log-log plot) however, after a threshold is reached for large datasets, memory utilization is flat.

Figure S2: Incremental PCA Pseudocode

Algorithm 1 Incremental PCA

```
Initialize:
  n_spectra_processed: int = 0
  processed_data_mean: vec = [0; feature_count]
  processed_data_variance: vec = [0; feature_count]
  processed_vt: mat = [ ]
  processed_s: vec = [ ]
Fit:
for each batch in dataset do
    n_spectra_batch: int = count_rows(batch)
    batch_mean: vec = col_mean(batch)
    incremental_mean: vec =
incremental_col_mean(batch, n_spectra_processed, processed_data_mean)
    batch −= batch_mean
    incremental_variance =
incremental_col_variance(batch, n_spectra_processed, processed_data_variance)
  if not first_batch then
      weight: float = sqrt((n_spectra_processed × n_spectra_batch) / (n_spectra_processed + n_spectra_batch))
      correction: vec = weight × (processed_data_mean − batch_mean)
      s_vt: mat = processed_vt × processed_s^T
      batch: mat = concatenate(s_vt, batch, correction)
  end if
    (u, s, vt) = SVD(batch)
    n_spectra_processed += n_spectra_batch
    processed_vt = vt
    processed_s = s
    processed_data_mean = incremental_mean
    processed_data_variance = incremental_var
end for
Transform:

for each batch in dataset do
    centered_batch = batch − processed_mean
    batch_component_values = centered_batch · processed_vt^T
end for
```

Figure S2: In this pseudocode, variables are of types integer (int), floating point values (float), 1-dimensional arrays (vec), or 2-dimensional arrays (mat). For more information please see the IPCA function in the scikit-learn library: https://scikit-learn.org/stable/auto_examples/decomposition/plot_incremental_pca.html
with source code at:
https://github.com/scikit-learn/scikit-learn/blob/main/sklearn/decomposition/_incremental_pca.py

Figure S3: Transformation step workflow

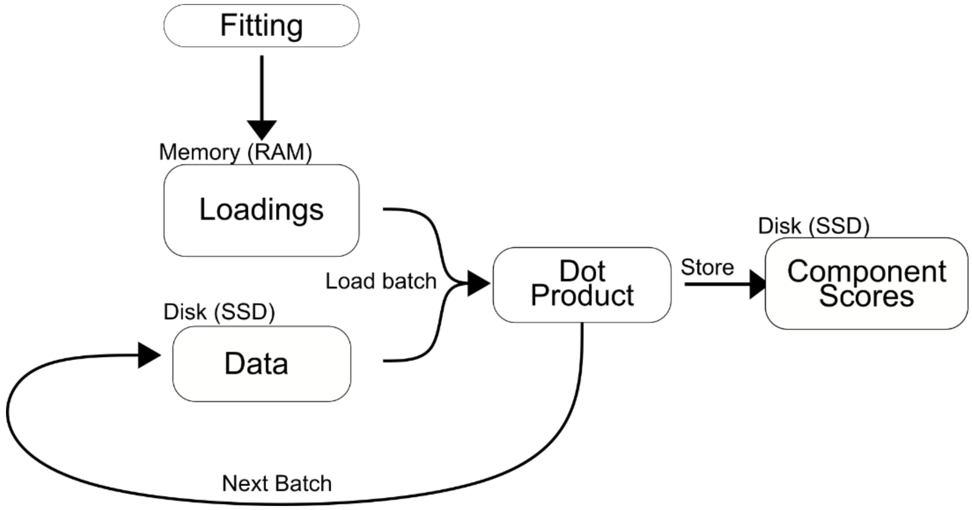

Figure S3: The “transforming” step of the IPCA workflow as shown in Figure 1 is expanded to show how the component scores are generated.

Table S1: Additional detail for benchmarking results

Table S1: Benchmarking Results

	Mouse Blader	Cancer Xenograft	Multiple Spinal Columns	Multiple Liver	Composed Surface
**Spectra**	34840	262144	571722	817207	256711021
**Mass Peaks**	199	310	415	573	33
**Datacube MB**	27.73264	325.05856	949.05852	1873.038444	33885.85477
**Original dataset formats**	.imzml and .ibd	Ionoptika J105 folder format	SCiLS lab .slx and .sbd files	TimsToF fleX	Raw TPX3CAM .tpx3 data
**Dataset GB (in original formats)**	0.0561	49.0873	1576.275	139.922	80.138
**Algorithm**			**Time (s)**		
**LipostarMSI**	252.100 ±6.655	6 735.080 ±56.128	†	†	†
**SCiLS Lab**	1.760 ±0.016	37.492 ±0.683	†	236.880 ±4.727	†
**R IRLBA C3**	15.98 ±0.137	180.548 ±1.129	488.025 ±2.442	963.839 ±8.769	†
**R IRLBA C30**	77.420 ±2.356	791.954 ±29.941	1963.283 ±61.856	3626.493 ±130.704	†
**6.5% Sampled**	0.0878 ±0.0120	0.873 ±0.00949	2.755 ±0.122	5.597 ±0.112	151.480 ±10.392
**Python IPCA**	0.168 ±0.001	2.379 ±0.073	7.481 ±0.097	16.999 ±0.437	252.435 ±2.850
**Python Unmodified IPCA**	0.396 ±0.014	5.126 ±0.042	16.370 ±0.166	38.346 ±1.055	‡
**Rust PCA**	0.138 ±0.001	3.470 ±0.011	10.858 ±0.037	23.526 ±0.506	‡
**Rust IPCA**	0.171 ±0.004	1.891 ±0.055	5.744 ±0.189	13.211 ±0.285	187.675 ±3.014
**Rust IPCA***	0. 152 ±0.001	1.584 ±0.007	4.903 ±0.038	11.173 ±0.054	140.866 ±2.345
**Algorithm**			**RAM (MB)**		
**LipostarMSI**	102.038 ±3.594	990.439 ±0.711	†	†	†
**SCiLS Lab**	567.863 ±7.633	5 866.755 ±0.867	†	32 900.138 ±1.848	†
**R IRLBA C3**	97.4 ±16.992§	314.3 ±84.149§	562.3 ±64.011§	977.9 ±117.774§	†
**R IRLBA C30**	89.8 ±16.719§	548.1 ±118.235§	1045.3 ±136.940§	1312.9 ±137.827§	†
**6.5% Sampled**	27.975 ±0.151	330.830 ±0.104	970.401 ±0.362	1911.357 ±0.282	35696.749 ±0.0615
**Python IPCA**	52.752 ±0.921	154.881 ±0.462	157.302 ±0.005	157.647 ±0.003	216.164 ±0.253
**Python Unmodified IPCA**	106.173 ±0.215	1 244.492 ±0.734	3 632.382 ±0.113	7 168.602 ±0.081	‡
**Rust PCA**	80.376 ±0.144	1 062.510 ±0.0421	3 006.021 ±0.014	5 779.466 ±0.023	‡
**Rust IPCA**	52.960 ±0.229	153.688 ±0.486	153.868 ±0.022	154.479 ±0.026	153.321 ±0.064
	53.214	615.210	1 732.863	2 451.293	2 491.707

Algorithm					
**Rust IPCA***	±0.076*	±4.514*	±72.405*	±0.650*	±61.206*
**Algorithm**	**RAM (MB) / Datacube MB**				
**LipostarMSI**	3.679	3.047	†	†	†
**SCiLS Lab**	20.476	18.048	†	17.565	†
**R IRLBA C3**	3.512§	0.967§	0.592§	0.522§	†
**R IRLBA C30**	3.238§	1.686§	1.101§	0.701§	†
**6.5% Sampled**	1.009	1.018	1.022	1.020	1.053
**Python IPCA**	1.902	0.476	0.166	0.0842	0.00638
**Python Unmodified IPCA**	3.828	3.829	3.827	3.827	‡
**Rust PCA**	2.898	3.269	3.168	3.086	‡
**Rust IPCA**	1.910	0.473	0.162	0.0825	0.00453
**Rust IPCA***	1.919	1.893	1.826	1.309	0.0735

*With a multithreaded transformation loop; †Unable to be loaded into software; ‡Caused out of memory error; § High standard deviation possibly due to R garbage collector

Table S1: Extended table version of Table 1 with a higher precision, standard deviation, added division section (where the RAM utilization of each algorithm is divided by the total datacube size for the data that was analyzed), added data size of the datacube and the original datasets (Note: datacube size often differs dramatically from dataset size as some datasets were cropped to remove unwanted regions, were not peak-picked, or were stored in a less efficient file format — this is especially observable in the Multiple Spinal Columns dataset which has ~1.6 TB of data files but is ~950 MB for the peak-picked datacube), and the addition the benchmarking of theunmodified, default scikit IPCA function using 64-bit float numbers, no batch selection (default is features × 5), and in-memory transformation without increments.

Figure S4: Batch size optimization threshold

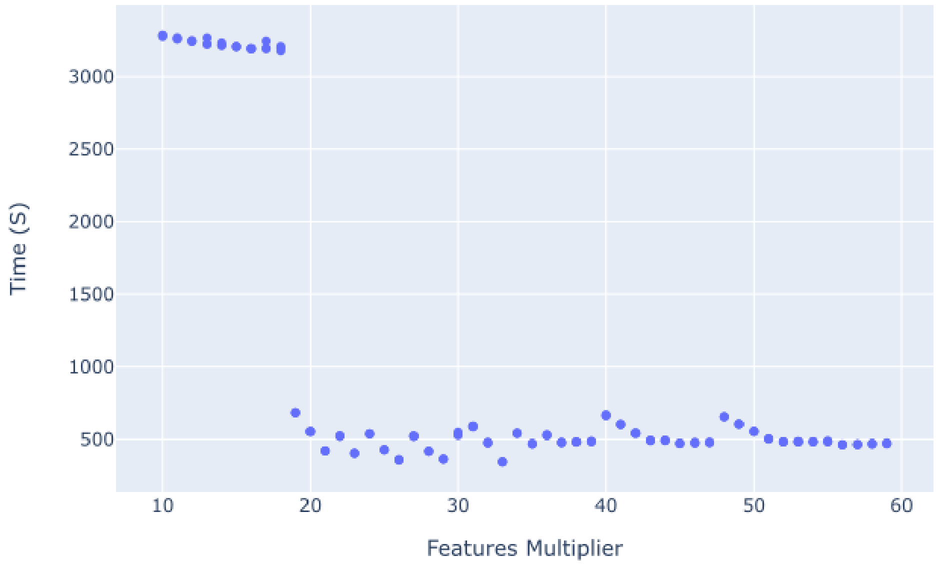

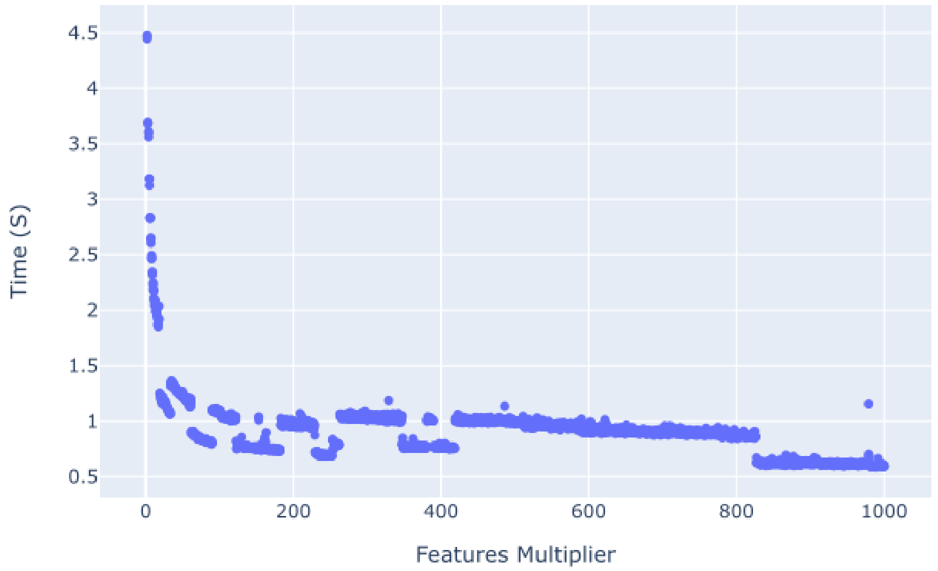

Figure S4: Top: Optimization of the batch size, for a simulated dataset containing 4096 features (peaks) and 1048576 samples (spectra). The x-axis is batch size as a multiplicative of the number of features. Notably there is a large drop in runtime after a batch size of features multiplied by 19.

Bottom: Optimization of the batch size, for a simulated dataset containing 32 features (peaks) and 1048576 samples (spectra). The x-axis is batch size as a multiplicative of the number of features. Notably there is a quick drop in runtime up to a batch size of features multiplied by ~19.

Figure S5: Batch size optimization threshold applied to a "real" dataset

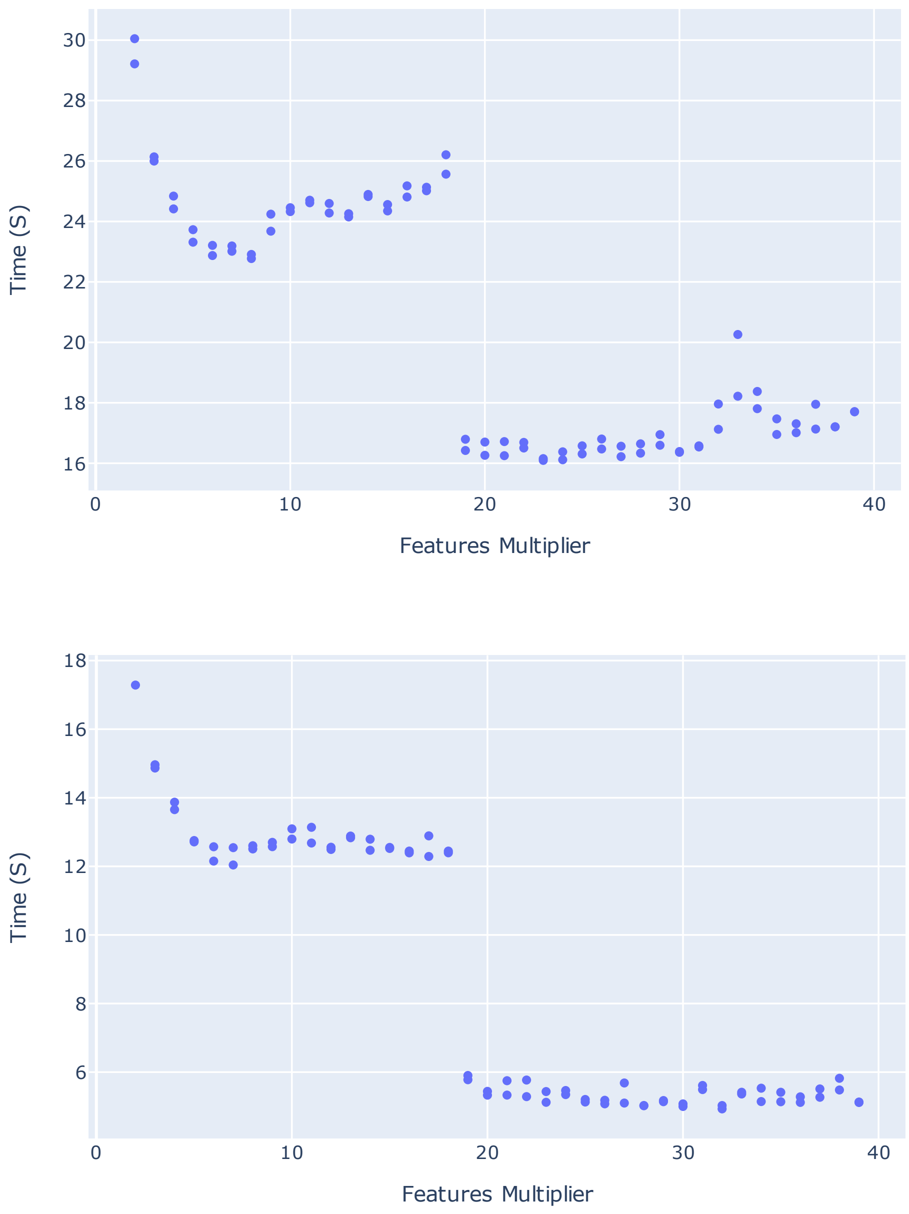

Figure S5: Top: Optimization of the batch size, on the multiple liver dataset while using the sequential Intel MKL.The x-axis is batch size as a multiplicative of the number of features. Notably there is a large drop in runtime after a batch size of features multiplied by 19.

Bottom: Optimization of the batch size, on the multiple livers dataset while using the multithreaded intel MKL. The x-axis is batch size as a multiplicative of the number of features. Notably there is a large drop in runtime after a batch size of features multiplied by 19.

Figure S6: Visual comparison of SCiLS and Rust IPCA results

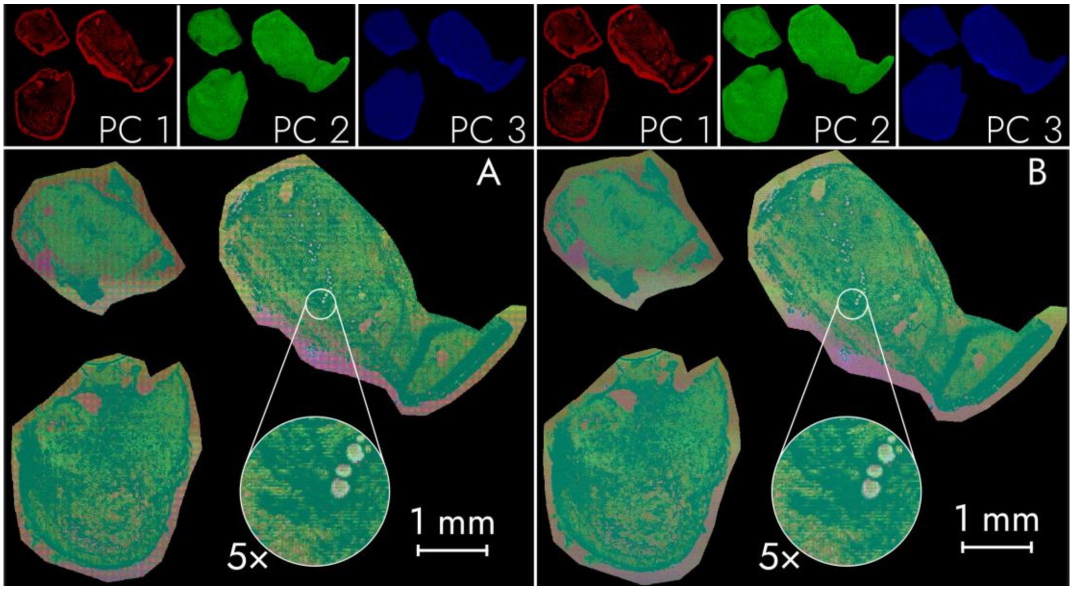

Figure S6: Hyperspectral visualization of the first three principal components for the Multiple Livers dataset processed by SCiLS Lab (A) and Rust IPCA (B). Principal components 1, 2, and 3 are visualized as linear gradients of red, green, and blue, respectively. The individual principal component images can be seen above their respective composites. Grid-like patterns present in A are artifacts of the rendering process SCiLS lab uses and not present in the 5× zoomed in data.

Table S2: MSE values of the comparison of algorithms compared against SCiLS Lab loading scores for the Multiple Livers dataset

Component	Rust IPCA	R IRLBA C30	6.5% Sampled Trial 1	6.5% Sampled Average of 9 trials	6.5% Sampled Standard Deviation of 9 trials
	Mean Square Error				
**1**	1.77E-10	1.77E-10	3.36E-09	2.74E-09	1.65E-09
**2**	7.87E-10	7.83E-10	6.53E-09	3.08E-08	3.01E-08
**3**	5.12E-10	5.04E-10	6.06E-08	6.85E-08	3.88E-08
**4**	6.84E-08	6.84E-08	1.68E-07	2.99E-07	2.22E-07
**5**	1.96E-07	1.96E-07	5.41E-07	7.70E-07	5.01E-07
**6**	3.21E-08	3.21E-08	1.45E-06	2.63E-05	5.09E-05
**7**	1.54E-07	1.54E-07	1.51E-06	2.65E-05	5.07E-05
**8**	1.45E-07	1.46E-07	4.36E-06	4.45E-06	3.67E-06
**9**	3.23E-07	3.23E-07	5.30E-06	7.27E-06	3.92E-06
**10**	1.20E-07	1.19E-07	1.86E-06	5.17E-06	4.12E-06
**11**	1.05E-06	1.05E-06	2.72E-06	3.09E-06	9.96E-07
**12**	2.59E-06	2.59E-06	4.21E-04	4.22E-04	1.09E-03
**13**	7.16E-07	7.21E-07	2.77E-03	6.61E-04	1.27E-03
**14**	3.74E-06	3.74E-06	3.56E-03	8.09E-04	1.56E-03
**15**	1.49E-05	1.49E-05	3.67E-03	1.04E-03	1.38E-03
**16**	8.13E-05	8.13E-05	2.07E-03	1.44E-03	1.57E-03
**17**	5.17E-07	5.18E-07	3.45E-03	2.75E-03	2.90E-04
**18**	8.22E-05	8.22E-05	2.58E-04	2.53E-03	1.80E-03
**19**	2.44E-05	2.44E-05	3.77E-05	2.19E-03	1.62E-03
**20**	3.95E-05	3.95E-05	4.16E-05	2.16E-03	1.63E-03
**21**	4.83E-05	4.72E-05	1.42E-05	1.63E-03	1.39E-03
**22**	4.66E-05	4.55E-05	1.41E-05	2.39E-03	1.62E-03
**23**	3.91E-06	3.88E-06	1.53E-05	1.40E-03	1.66E-03
**24**	5.05E-05	4.77E-05	1.07E-05	8.47E-04	1.05E-03
**25**	5.09E-05	4.82E-05	2.48E-05	1.35E-03	1.48E-03
**26**	5.66E-05	5.65E-05	9.74E-05	1.32E-03	1.75E-03
**27**	6.28E-05	6.28E-05	2.48E-04	1.15E-03	1.39E-03
**28**	5.41E-05	5.42E-05	1.18E-04	1.13E-03	1.48E-03
**29**	5.42E-05	5.42E-05	8.41E-05	8.13E-04	1.41E-03
**30**	9.76E-06	9.74E-06	2.64E-05	7.96E-04	1.48E-03

Table S3: r^2 values of the comparison of algorithms compared against SCiLS Lab loading scores for the Multiple Livers data

	Rust IPCA	R IRLBA C30	6.5% Sampled Trial 1	6.5% Sampled Average of 9 trials	6.5% Sampled Standard Deviation of 9 trials
**Component**			r^2		
**1**	0.999999898	0.999999898	0.9999980	0.999998421	9.56077E-07
**2**	0.999999549	0.999999552	0.9999960	0.999982573	1.71056E-05
**3**	0.999999707	0.999999712	0.9999660	0.999961797	2.17141E-05
**4**	0.999959104	0.999959114	0.9999010	0.999832173	0.000115179
**5**	0.999874426	0.999874431	0.9996540	0.999514634	0.000320739
**6**	0.999981802	0.999981821	0.9992970	0.986412813	0.026422751
**7**	0.999903421	0.999903449	0.9990500	0.983966721	0.030341974
**8**	0.999916716	0.999916319	0.9975170	0.997444492	0.002117307
**9**	0.999814066	0.999813714	0.9969460	0.995862943	0.002256534
**10**	0.999929935	0.999930335	0.9989160	0.996985477	0.002402707
**11**	0.999400973	0.999400906	0.9984430	0.998246742	0.000558363
**12**	0.998481983	0.998481435	0.7689580	0.85901284	0.329844092
**13**	0.999584118	0.999581607	0.0406762	0.774764506	0.424316106
**14**	0.997860172	0.997859513	0.0000969	0.767783712	0.435295545
**15**	0.991564805	0.991577981	0.0029635	0.631828333	0.385771477
**16**	0.953770484	0.953772073	0.1656670	0.523122187	0.375985528
**17**	0.999702976	0.99970218	0.0000312	0.050240439	0.024387373
**18**	0.953438518	0.95342665	0.8591400	0.312792283	0.460916269
**19**	0.986091943	0.98609796	0.9783670	0.33248641	0.483008535
**20**	0.977343022	0.977343094	0.9764370	0.33980159	0.475996462
**21**	0.972048519	0.972702356	0.9917410	0.425877235	0.42337443
**22**	0.973956133	0.974590108	0.9919960	0.291522869	0.437824673
**23**	0.9977881	0.997808397	0.9911900	0.558962753	0.513961484
**24**	0.971764792	0.973318987	0.9938780	0.657972715	0.298104203
**25**	0.970460656	0.972011535	0.9858590	0.533142774	0.415280665
**26**	0.969218367	0.969274053	0.9470780	0.614432167	0.419540377
**27**	0.962297934	0.96227187	0.8548510	0.588409489	0.4170488
**28**	0.969823452	0.969755012	0.9364420	0.617214309	0.453443293
**29**	0.970562183	0.970546598	0.9548990	0.734542307	0.414830328
**30**	0.994520086	0.994529974	0.9849960	0.75567642	0.426696613
